# Patient-reported impact of spondyloarthritis on work disability and working life: the ATLANTIS survey

**DOI:** 10.1186/s13075-016-0977-2

**Published:** 2016-04-01

**Authors:** Roberta Ramonda, Antonio Marchesoni, Antonio Carletto, Gerolamo Bianchi, Maurizio Cutolo, Gianfranco Ferraccioli, Enrico Fusaro, Salvatore De Vita, Mauro Galeazzi, Roberto Gerli, Marco Matucci-Cerinic, Giovanni Minisola, Carlomaurizio Montecucco, Raffaele Pellerito, Fausto Salaffi, Giuseppe Paolazzi, Piercarlo Sarzi-Puttini, Raffaele Scarpa, Gianfilippo Bagnato, Giovanni Triolo, Guido Valesini, Leonardo Punzi, Ignazio Olivieri, ATLANTIS study group

**Affiliations:** Rheumatology Unit, Department of Medicine DIMED, University of Padova, Via Giustiniani, 2, Padova, 35128 Italy; Division of Rheumatology, Day Hospital Unit, Istituto Ortopedico G. Pini, Milano, Italy; Rheumatology Unit, University of Verona, Verona, Italy; Department of Locomotor System, Division of Rheumatology, ASL3-Azienda Sanitaria Genovese, Arenzano, Genova, Italy; Research Laboratory and Academic Division of Clinical Rheumatology, Department of Internal Medicine, IRCCS A.O.U. San Martino-IST, University of Genova, Genova, Italy; Division of Rheumatology, Institute of Rheumatology & Affine Sciences, Catholic University School of Medicine, Roma, Italy; Rheumatology Unit, Città della Salute e della Scienza, University Hospital of Torino, Torino, Italy; Rheumatology Clinic, Department of Medical and Biological Sciences, Azienda Ospedaliero-Universitaria S. Maria della Misericordia, Udine, Italy; Research Center of Systemic Autoimmune and Autoinflammatory Diseases, University of Siena, Siena, Italy; Rheumatology Unit, Department of Medicine, University of Perugia, Perugia, Italy; Department of Experimental and Clinical Medicine, Division of Rheumatology, Azienda Ospedaliero-Universitaria Careggi (AOUC), University of Firenze, Firenze, Italy; Division of Rheumatology, San Camillo Hospital, Roma, Italy; Department of Rheumatology, University of Pavia, IRCCS San Matteo Foundation, Pavia, Italy; Rheumatology Unit, Ospedale Mauriziano, Torino, Italy; Rheumatology Department, Polytechnic University of the Marche Region, Jesi, Italy; Department of Rheumatology, Santa Chiara Hospital, Trento, Italy; Rheumatology Unit, L. Sacco University Hospital, Milano, Italy; Rheumatology Research Unit, Department of Clinical and Experimental Medicine, University Federico II, Napoli, Italy; Department of Clinical and Experimental Medicine, University of Messina, Messina, Italy; Department of Internal Medicine, Rheumatology Unit, University of Palermo, Palermo, Italy; Rheumatology Unit, La Sapienza University of Roma, Roma, Italy; Rheumatology Department, San Carlo Hospital, Potenza, Italy

**Keywords:** Spondyloarthritis, Survey, Absenteeism, Presenteeism, WPI

## Abstract

**Background:**

The aim was to establish how patients experience the impact of spondyloarthritis (SpA) on work disability and working life.

**Methods:**

The survey was performed in 17/20 regions in Italy (1 January to 31 March 2013). A multiple-choice questionnaire was published on the official website of the sponsor - the National Association of Rheumatic Patients (ANMAR) - and hard-copies were distributed at outpatient clinics for rheumatic patients.

**Results:**

Respondents (n = 770) were of both sexes (56 % men), educated (62 % at high school or more), of working age (75 % aged ≤60 years), and affected by SpA. The most common types diagnosed were ankylosing spondylitis (AS) (39 %) and psoriatic arthritis (PsA) (36 %). Respondents were working full-time (45 %), part-time (8 %) or had retired (22 %); 15 % were unemployed (for reasons linked to the disease or for other reasons, students or housewives). Patients reported disability (39 %), were receiving disability benefits (34 %), were experiencing important limitations that were hindering their professional development/career (36 %) and some had to change/leave their job or lost it because of SpA (21 %). Employed respondents (n = 383) had worked on average 32.2 h in the last 7 days. More hours of work were lost over the last 7 days due to SpA (2.39 h vs 1.67 h). The indirect costs of the disease amounted to €106/week for patients reporting well-being/good physical conditions/improvement and €216/week for those reporting permanent impairment.

**Conclusions:**

Most patients were in the midst of their productive years and were experiencing considerable difficulties in carrying out their job because of the disease: half of them reported disability and one third were experiencing important limitations in their career perspective.

**Electronic supplementary material:**

The online version of this article (doi:10.1186/s13075-016-0977-2) contains supplementary material, which is available to authorized users.

## Background

Major advances have occurred in the diagnosis and classification of spondyloarthritis (SpA) over the last 10 years, especially since spinal inflammation was demonstrated on magnetic resonance imaging (MRI) in the absence of radiographic evidence of change [1]. The changes were formalized in the revised versions of the Assessment of SpondyloArthritis International Society (ASAS) classification criteria for axial (ax) [2] and peripheral SpA [3]. Thanks to these criteria, patients with axial SpA without radiographic evidence of sacroiliitis can now be classified as having non-radiographic axSpA, a form which does not appear to differ significantly from radiographic axSpA in terms of the main clinical manifestations, disease activity, pain, quality of life and response to anti-tumor necrosis factor (anti-TNF) therapy [4–6]. Differences between patients with non-radiographic and radiographic axSpA have been found in age, symptom duration, human leukocyte antigen (HLA)-B27 positivity [7] and gender distribution. Although less inflammation (i.e., lower C-reactive protein and less evidence of spinal inflammation on MRI) and less impairment in spinal mobility have been observed in non-radiographic axSpA than in the axSpA, the two conditions are associated with a similar burden in terms of disease activity, physical function and health ratio quality of life (HR-QoL) impairment [8]. ASAS classification criteria are also important because they enable detection of early SpA, so that treatment can be introduced earlier than in the past when the average delay in diagnosis was about 10 years [9]. Of course, former classification criteria for specific subtypes of SpA, such as the modified New York Criteria for ankylosing spondylitis (AS) [10] or Classification Criteria for Psoriatic Arthritis (CASPAR) for psoriatic arthritis (PsA) [11] remain valid to classify particular forms of the disease.

Before the introduction of the ASAS criteria, the global prevalence of SpA was calculated to be approximately 1 %, with notable variability, ranging from 0.01 % in Japan to 2.5 % in Northern Arctic populations. The recognition of non-radiographic axSpA will have a considerable impact on the prevalence of SpA, because many patients who had an unclassifiable axSpA can now be classified according to the ASAS criteria [1, 8].

The increase in the prevalence of SpA has notable socioeconomic implications. By definition, SpA is a disease that occurs in young adults at the peak of their productive lifespan [2]. Moreover, it is associated with a considerable burden in terms of restrictions in activities of daily living [12, 13], reduction in health-related quality of life (HR-QoL) [13, 14] and in work productivity, and in terms of unemployment [15–17].

The reduction in work productivity is an important component of the indirect costs of SpA, which are typically calculated in terms of absenteeism and presenteeism using tools such as the Work Productivity and Activity Impairment Questionnaire in AS (WPAI) [18]. Absenteeism is defined as the quantity of sick leave either specifically due to the disease itself or, in the event that it is difficult to distinguish between sick leave due to the disease itself and sick leave due to concomitant or incident diseases, the total amount of sick leave expressed as days in a year or hours per week. Presenteeism is defined as patient-reported reduced productivity at work [18]. Caution is required in the extrapolation of data from one country to another, because findings on work productivity and unemployment are affected by a number of local factors, primarily the local unemployment/disability benefit system [19].

We carried out a survey in Italy called Atlantis (an underwater world that we would like to bring to light), which was designed to establish how the diagnostic work-up and management of SpA in Italy is experienced by the patients, and assess the impact of SpA on daily living, personal relationships, work disability and working life. This paper reports findings on work disability and working life.

## Methods

### Study characteristics

This was a cross-sectional survey sponsored by the Italian National Association of Rheumatic Patients (ANMAR). It was carried out in 17 out of the 20 regions in Italy from 1 January to 31 March 2013 by Doxa-Pharma, a company experienced in carrying out surveys in the healthcare field, under the guidance of an experienced rheumatologist (RR, author), who was the scientific coordinator of the project. According to Italian regulations, the study did not require written informed consent because the questionnaire was completed anonymously. Additional sensitive data were not collected.

### Patients and methods

The survey was conducted on patients with a defined diagnosis of SpA made by a rheumatologist and the questionnaire was drafted with five sections: (1) personal details and diagnosis of the patient, including information on work status and any welfare benefits granted; (2) the diagnostic work-up; (3) follow up; (4) impact of the disease on quality of life; and (5) impact on working life, including absenteeism and presenteeism. We considered the indirect costs of the disease in terms of sick leave, which was defined as the weekly cost due to the hours of absence from the job because of the disease, and in terms of presenteeism, which was defined as the quantification of the weekly cost due to the lower job performance of the patients because of the disease. The sum of these two indices, termed the time loss, was defined as the weekly income that was not produced (indirect cost), considering the Italian weekly per capita income in euros in 2012 divided by the standard 44 weeks of work. The multiple-choice questions focused on work status and working life (see Additional file 1 for all questions in detail). Data were collected through two parallel channels: (1) the survey questionnaire was published on the web, with a link placed at the official ANMAR website (www.anmar-italia.it), to be completed by patients who were willing to do so, and (2) hard copies of the survey questionnaire were distributed throughout the Italian regions of interest by ANMAR volunteers, with the support of specialists at the hospitals with clinics dedicated to the management of rheumatic patients.

### Data analysis

The data were analyzed descriptively. For the open questions we read all the answers and encoded various items. Data were expressed in terms of frequency and averages. Electronic software (SAS and DIANA) was used to perform an interpretative reading of these data. Collected data were analyzed in relation to sociodemographic variables (sex, age, area of residence) and the type of specific SpA disease.

## Results

A total of 770 subjects took part in the survey. Their main features are summarized in Table 1.

**Table 1:** Main features of survey respondents

Sociodemographic characteristics	Respondents, %
Gender	
Male	56 %
Female	44 %
Age, years	
≤30	8 %
31–40	17 %
41–50	26 %
51–60	27 %
61–70	17 %
>70	4 %
Not specified	1 %
Education	
Degree/Master’s degree	17 %
High school	45 %
Junior high school	28 %
Primary school	8 %
Not specified	2 %
Marital status	
Married/living with partner	64 %
Single	20 %
Divorced/separated/widow(er)	12 %
Not specified	4 %
Residence	
North-West	24 %
North-East	26 %
Central regions	26 %
South and Islands	24 %
Diagnosis	
Ankylosing spondylitis	41 %
Psoriatic arthritis	39 %
Spondyloartritis with inflammatory bowel disease	10 %
Undifferentiated spondyloarthritis	6 %
Reactive arthritis	4 %

**Fig. 1:**
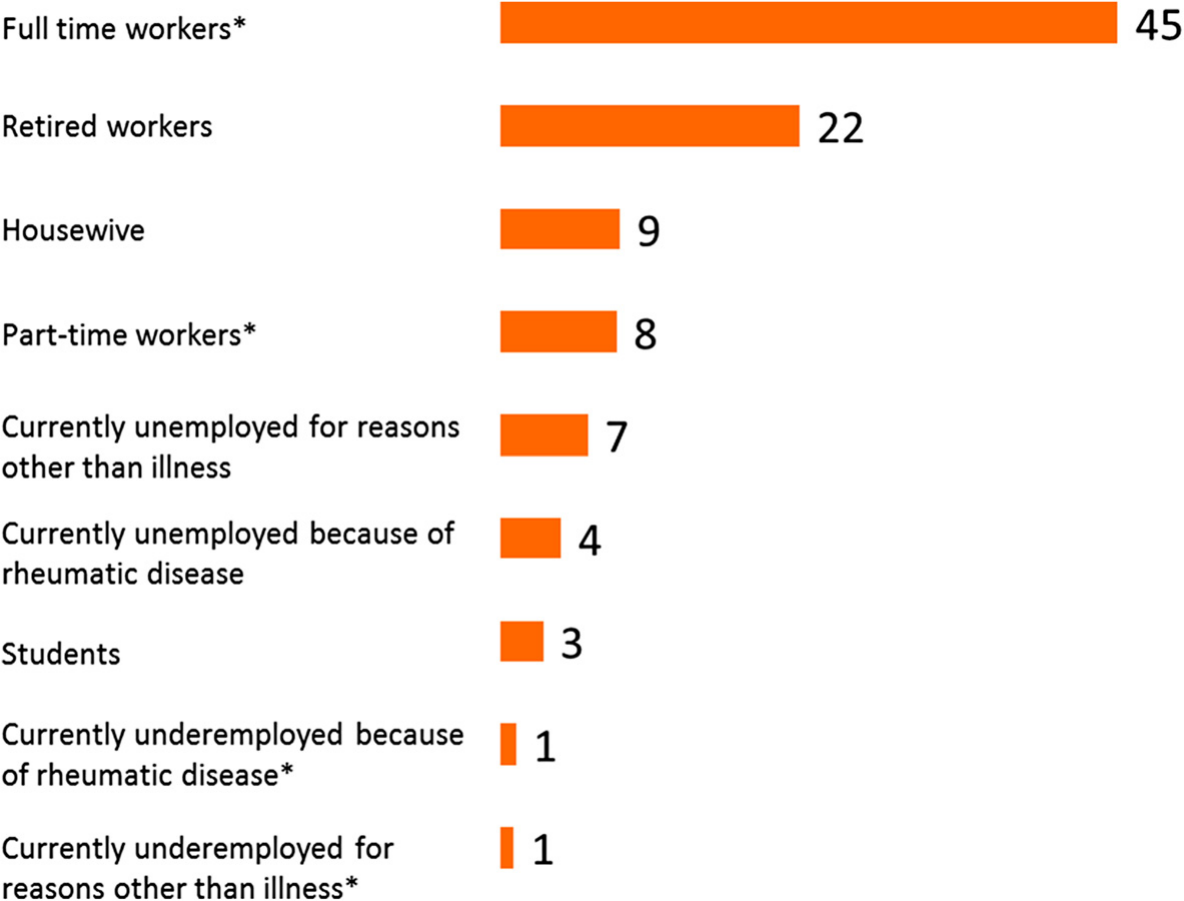
Main work status in patients with spondyloarthritis

More respondents were men (56 %). Most of them were married or living with a partner (64 %), were of working age (78 % aged ≤60 years; mean age = 50 years) and educated (62 % had completed high school or had a university degree). Their distribution throughout Italy was homogeneous, ranging from 24–26 % in the four main Italian geographical areas. The subjects were suffering from AS (41 %), PsA (39 %) SpA with inflammatory bowel diseases (IBD) (10 %), undifferentiated SpA (6 %) and reactive arthritis (4 %) (Fig. 1). Many patients reported that they had contacted other specialists after the diagnosis: ophthalmologists 50 %, cardiologists 45 %, dermatologists 43 %, gastroenterologists 37 %, psychologists 10 %, specialists in infectious diseases 8 %, pulmonologists 2 %, neurologists 2 %, gynecologists 1 %, endocrinologists 1 %, and neurosurgeons 1 % [20].

Slightly below half of the respondents were working either full-time (45 %) or part-time (8 %). Nearly one fourth had retired (22 %) and 15 % were unemployed (for reasons linked to SpA in 4 % of the cases; the remaining were students, housewives or individuals who were unemployed due to reasons other than SpA) (Fig. 1). Half of the respondents (49 %) reported that they were disabled (Table 2) and had to cancel or postpone commitments because of their disease (50 %) and 13 % declared that they had to do this at least half of the time. This depended on symptom control.

**Table 2:** Impact on working life

Reported consequences of SpA	Respondents, %
Disability	49 %
Receives disability benefits	34 %
Has applied for disability benefits	8 %
Cannot work full-time, but does not meet criteria for disability benefit	7 %
Has to cancel or postpone work commitments because of SpA	50 %
Does not apply	27 %
Never	23 %
Sometimes	37 %
About half the time	7 %
Most of the time	4 %
All the time	2 %
Limitations to work projects or career in general because of SpA	48 %
Does not apply	20 %
None	12 %
Negligible	20 %
Minor limitations	12 %
Tangible limitations	15 %
Major limitations	21 %
Had to change/leave a job or lost a job because of SpA	21 %
Yes	21 %
Does not apply	24 %
No	55 %
Has felt discriminated at work because of SpA	14 %
Yes	14 %
Does not apply	31 %
No	55 %
Fewer raises in salary because of SpA	14 %
Does not apply	44 %
Not at all	36 %
Negligible	6 %
A little	4 %
Quite a bit	7 %
A lot	3 %

*SpA* spondyloarthritis

**Fig. 2:**
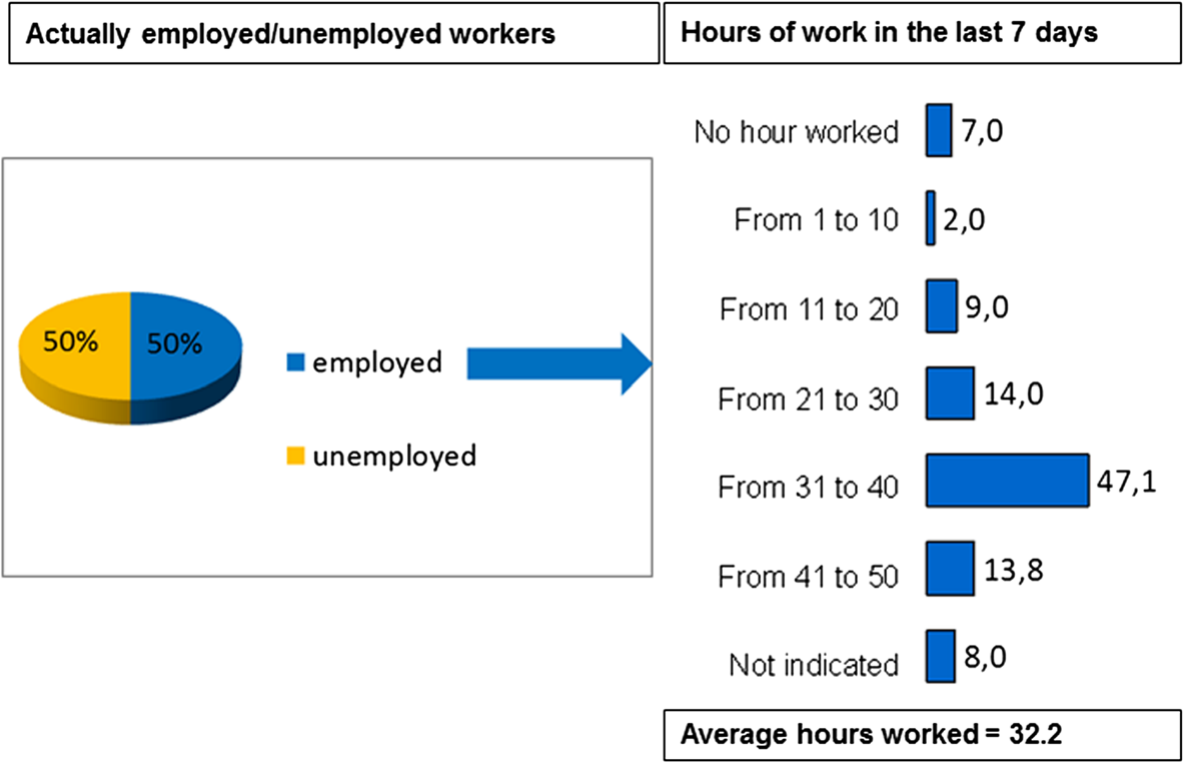
Results (employed/unemployed workers) by the survey

One third of patients (36 %) experienced important limitations that hindered their professional development/career. Of these, 19 % had controlled symptoms, 32 % were improving and 50 % had uncontrolled symptoms. One in five patients actually had to change or leave their job or had actually lost it because of SpA (21 %). Discrimination may have played a role in some cases, because 14 % of subjects reported this, and another 14 % believed that SpA had had an impact on their salary (Table 2). The impact on salary also depended on symptom control: 12 % of the patients with controlled symptoms believed that their disease had had a negative effect on their salary compared to 20 % with uncontrolled symptoms. The 383 respondents (49.7 % of the total sample) who were employed had worked on average 32.2 h in the last 7 days. Approximately 60 % had actually worked full-time (31 h or more) (Fig. 2).

Questions about absenteeism revealed that more hours had been lost over the previous 7 days due to SpA (on average 2.39 h vs 1.67 h), than for other reasons such as vacation or family commitments. The hours of absence because of SpA amounted to 59 % of the hours of absence from the work place and to 7 % of weekly working hours. The time loss cost was €216 per week for the patients reporting permanent impairment and €106 euros for the patients who reported well-being or an improvement in their physical condition.

## Discussion

This survey spearheaded by ANMAR has shown that patients with SpA experience major disadvantages in their working life: one half of the respondents claimed that they were disabled, and one half reported that SpA interfered significantly with their work commitments; SpA was the main source of absenteeism and presenteeism. What is more, the survey identified a number of issues that, to our knowledge, have not been investigated to date: one half experienced restrictions in their work prospects and career, 21 % had had to change/leave a job or had lost a job because of SpA, and 14 % had experienced discrimination at the workplace.

The sample was representative of the SpA population in terms of gender, age and diagnosis, [1, 21, 22]. Participation from all four main geographical areas of Italy was almost the same for each (24–26 %). The collection of data both via the website and distribution at hospital outpatient clinics ensured that there was no pre-selection of computer-literate patients [23]. Even so, the survey did include patients who were more educated than average, as only 28 % of respondents had not continued after junior high school, whereas the national average in 2013 was 44 % [23]; this is probably due to the fact that the elderly (a less educated generation) were under-represented in the sample (patients over 60 years of age represented 21 % vs the 27.2 % in the general population [24].

The employment rate was slightly lower than in the general population, being 53 % instead of the expected rate of 58 %, according to the official employment statistics in the general population in Italy in 2013 [25], after correction for the higher percentage of male individuals in the survey than in the general population. Discrimination may have played a role in some cases; in fact, 14 % of the patients reported having the impression of being treated differently from other colleagues due to their rheumatic disease. The proportion of SpA patients receiving disability benefits was nearly five times higher than the proportion in the general population (34 % vs 7.3 %) [24, 26].

One of our study limitations was the use of a non-validated questionnaire. However, our findings on work disability and reduction in productivity, both in terms of absenteeism and presenteeism, are consistent with similar published studies, which all report that SpA is associated with disability and reduction in productivity. Nevertheless, comparisons between different studies is difficult because of the differences in patient populations (e.g., the proportions with manual vs non-manual occupations, proportions with non-SpA-related comorbidities), and in the definition of endpoints (e.g., sick leave may be total or restricted to leave due to SpA), and, above all, differences in local employment and benefit systems [25]. The mean total sick leave was 4 hours per week, which amounts to 26 days of sick leave a year, which is well within the range reported in the literature mentioned above.

In our results we also included an assessment of indirect costs, in terms of time loss, which is the sum of sick leave and presenteeism costs. In this study time loss cost was €216 per week for the patients with permanent impairment and €106 euros for the patients who were well or claimed that their physical conditions had improved.

Another limitation of this survey is the lack of information on disease duration. However, it has been shown that productivity is substantially reduced, even in patients with early SpA (mean disease duration 4.8 years) [27]. This evidence is cause for major concern, because the onset of the disease is in young adulthood and it therefore results in limitations during patients’ most productive years. Indeed, this prospect was emphasized by the fact that nearly half of the respondents had the perception of a loss in their work prospects.

To our knowledge, this is the first survey that documents the perception of limitations in work prospects and career, and discrimination at the workplace. A number of factors have been found to be associated with work disability and loss of productivity at work. Unemployment is associated with older age, social deprivation, longer disease duration, functional impairment, and depression, whereas absenteeism is associated only with disease activity and depression, and presenteeism with older age, disease activity, anxiety, and depression [28]. In particular, in a cross-sectional self-reported postal survey examining 1447 Swedish respondents with SpA, presenteeism was associated with a series of disease activity and quality of life measures, including the Bath Ankylosing Spondylitis Activity Index (BASDAI), the Bath Ankylosing Spondylitis Functional Index (BASFI), the EuroQoL (EQ)-5D pain and symptom, and the ASAS scores [17]. Findings were similar within the more rigorous context of a randomized, controlled clinical trial (ATLAS) in which there was significant correlation between the Short Form (SF)-36/Ankylosing Spondylitis Quality of Life (ASQoL), BASDAI/BASFI,/Health Utility Index (HUI)-3 scores and WPAI-Specific Health Problem (SHP) scores, and between the patient’s and the physician’s global assessment of disease activity and WPAI-SHP scores [29]. The correlation between the level of disease activity and work productivity suggests that treatments able to control disease activity would also reduce disability and loss of productivity [30–34].

## Conclusions

As SpA impacts productivity mainly in the prime of patients' working lives, it is important to increase public awareness, so that patients will be in a position to seek timely, appropriate care, which will undoubtedly optimize their diagnostic and therapeutic outcome.

## Additional file

Additional file 1: Anonymous questionnaire (multiple choice questions). (PDF 56 kb)

## Abbreviations

ANMAR: Italian National Association of Rheumatic Patients
AS: ankylosing spondylitis
ASAS: Assessment of SpondyloArthritis International Society
ASQoL: Ankylosing Spondylitis Quality of Life
Ax: axial
BASDAI: Bath Ankylosing Spondylitis Activity Index
BASFI: Bath Ankylosing Spondylitis Functional Index
EQ-5D: EuroQoL-5D
HR-QoL: health-related quality of life
HUI-3: Health Utility Index scores
IBD: inflammatory bowel disease
MRI: magnetic resonance imaging
PsA: psoriatic arthritis
SF-36: Short Form-36
SpA: spondyloarthritis
TNF: tumor necrosis factor
WPAI: Work Productivity and Activity Impairment Questionnaire
