# Supplementary material for: Patient-reported impact of spondyloarthritis on work disability and working life: the ATLANTIS survey
Source: Arthritis Res Ther. 2016 Apr 1;18:78. doi: 10.1186/s13075-016-0977-2 (PMC4818386; doi:10.1186/s13075-016-0977-2)
Supplement: Additional file 1: — Anonymous questionnaire (multiple choice questions). (PDF 56 kb) [file 13075_2016_977_MOESM1_ESM.pdf]

Additional file 1. Anonymous questionnaire (multiple choice questions)

1. Specification of work status (full-time employment; part-time employment; unemployed /underemployed on account of reasons other than SpA or because of SpA; retired; housewife; student).
2. Which of the following conditions best describes your situation? I cannot work full-time any more, but I do not qualify for disability benefits; I have applied for disability benefits; I have disability benefits; none of the above.
3. During current or recent work activities, how many times have you had to cancel or postpone a commitment because of SpA? Does not apply to me; never; sometimes, about 50% of the time; most of the time; always
4. Have you ever had to leave /change your job or have you ever lost your job because of SpA? Yes; no; does not apply to me
5. Up to now has SpA ever restricted your work / career plans in general (e.g. limitation in performing certain tasks or practicing because of particular manifestations of the disease)? Does not apply to me; not at all; very little; not much; quite a bit; a lot
6. Have you ever felt that you were treated differently at work because you are a rheumatic patient? Yes; no; does not apply
7. Do you believe that SpA has had an impact on your salary (e.g. less raises than your healthy colleagues)? Does not apply to me; not at all; very little; not much; quite a bit; a lot
8. In the last 7 days, how many hours did you work? None; 1-10 h; 11-20h; 21-30h; 31-40h; 41-50h; not specified
9. In the last 7 days, how many hours of work did you miss for reasons related to SpA? None; 1-5 h; 6-10h; 11-15h; 16-20h; >20h; not specified
10. In the last 7 days, how many hours of work did you miss for reasons other than SpA? None; 1-5 h; 6-10h; 11-15h; 16-20h; >20h; not specified
11. How do you usually feel at work? Well; unwell
